# MiR-7 Triggers Cell Cycle Arrest at the G1/S Transition by Targeting Multiple Genes Including Skp2 and Psme3

**DOI:** 10.1371/journal.pone.0065671

**Published:** 2013-06-06

**Authors:** Noelia Sanchez, Mark Gallagher, Nga Lao, Clair Gallagher, Colin Clarke, Padraig Doolan, Sinead Aherne, Alfonso Blanco, Paula Meleady, Martin Clynes, Niall Barron

**Affiliations:** 1 National Institute for Cellular Biotechnology, Dublin City University, Dublin, Ireland; 2 Conway Institute, University College Dublin, Dublin, Ireland; CNRS UMR7275, France

## Abstract

MiR-7 acts as a tumour suppressor in many cancers and abrogates proliferation of CHO cells in culture. In this study we demonstrate that miR-7 targets key regulators of the G1 to S phase transition, including Skp2 and Psme3, to promote increased levels of p27^KIP^ and temporary growth arrest of CHO cells in the G1 phase. Simultaneously, the down-regulation of DNA repair-specific proteins via miR-7 including Rad54L, and pro-apoptotic regulators such as p53, combined with the up-regulation of anti-apoptotic factors like p-Akt, promoted cell survival while arrested in G1. Thus miR-7 can co-ordinate the levels of multiple genes and proteins to influence G1 to S phase transition and the apoptotic response in order to maintain cellular homeostasis. This work provides further mechanistic insight into the role of miR-7 as a regulator of cell growth in times of cellular stress.

## Introduction

The discovery of miRNAs has changed the perception of post-transcriptional regulation, adding another degree of control to the molecular mechanisms of most if not all cellular and signalling pathways [1], [2]. MiRNAs are involved in complex networks with other miRNAs, mRNA targets and transcription factors [3] and are highly conserved between species [4], [5]. In contrast to proteins, miRNAs do not compete with the translational machinery of the host cell and they also have the potential to regulate hundreds of targets [6]. This makes them attractive potential engineering tools for improving recombinant protein production by CHO cells. In general, miRNAs are transcribed through RNA polymerase II. Their processing into small double-stranded molecules occurs after a two-step cleavage by RNase III-like enzymes. The guide strand of the miRNA is loaded into the miRNA-induced silencing complex (miRISC) [7], [8], leading to translation repression and/or mRNA destabilisation in mammalian cells [9], [10], [11]. Down-regulation of miR-7 expression has been reported in many cancers including breast [12], pancreatic [13], glioblastoma [14], lung [15] and tongue squamous cell carcinoma [16]. During embryogenesis, miR-7 plays a pivotal role in maintaining homeostasis in Drosophila during episodes of environmental flux [17], [18]. Like most miRNAs, the exact role of miR-7 depends not only on the cell type but also on other conditions. Although several recent publications have addressed the role of miR-7, much remains to be elucidated to fully unravel the entire network of its interactions. Recently, we showed that transfection of miR-7 induced transient cell growth arrest over a period of 96 hrs while maintaining high cell viability in CHO cells [19]. This phenotype mimics somewhat the impact of reducing CHO culture temperature during the production of recombinant therapeutic proteins in the Biopharmaceutical industry. In this study, we attempt to identify the genes and proteins targeted by miR-7 which may trigger arrest in the G1 phase of the cell cycle while avoiding apoptosis-dependent programmed cell death.

## Results

### Up-regulation of miR-7 induces transient arrest in the G1 phase of the cell cycle without promoting apoptosis

**Figure 1:**
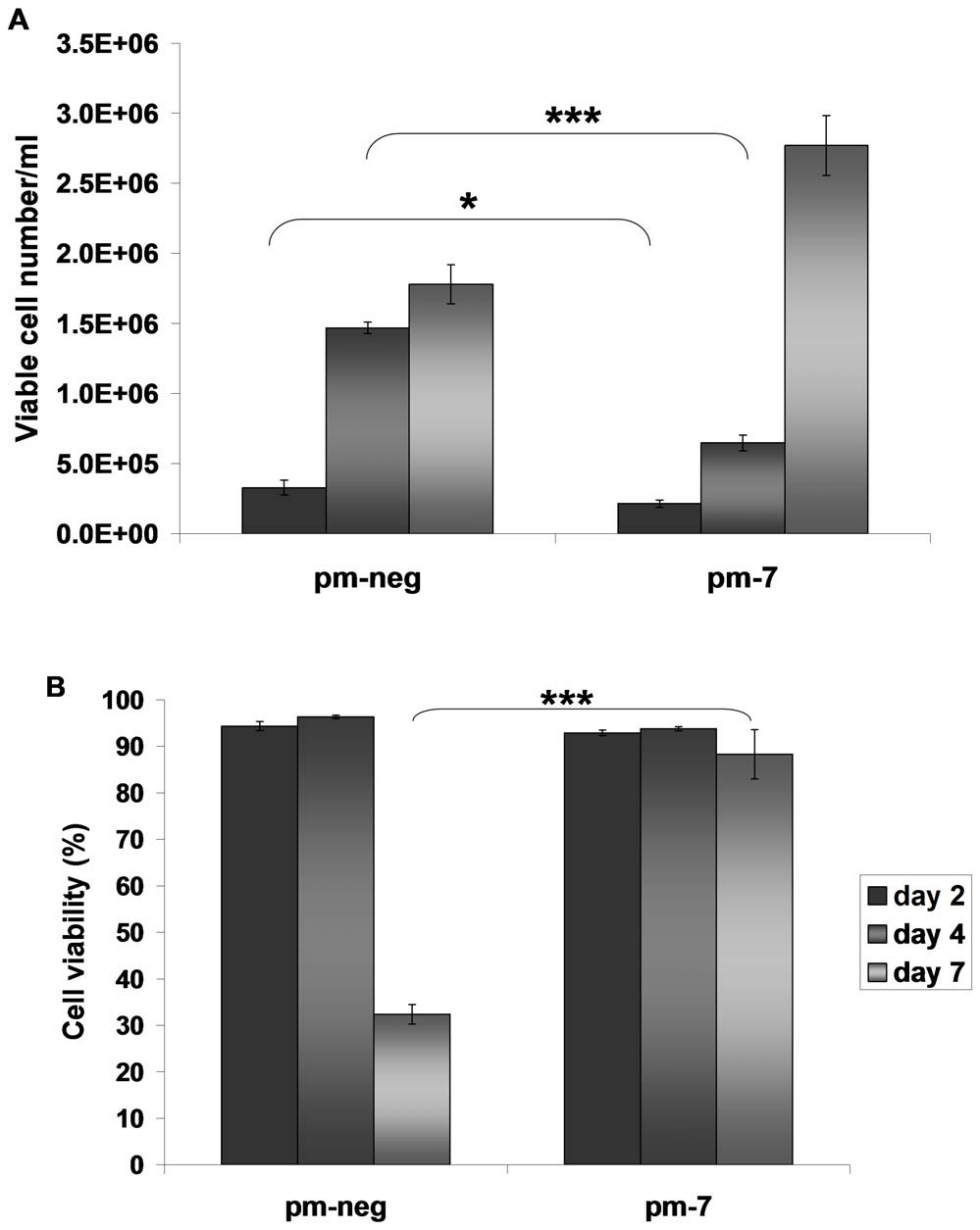
Impact of miR-7 transfection on cell growth (A) and cell viability (B). Mimics of miR-7 (pm-7) or non-specific mimic controls (pm-neg) were transfected using 2 µl of NeoFx™ in CHO cells at a concentration of 50 nM. Cells were stained with GuavaViacount reagent to assess cell density and cell viability at day 2, day 4 and day 7. Standard deviations represent biological triplicates. Statistics were evaluated with a Student's t-test.*: p-value<0.05; ***: p-value<0.001.

**Figure 2:**
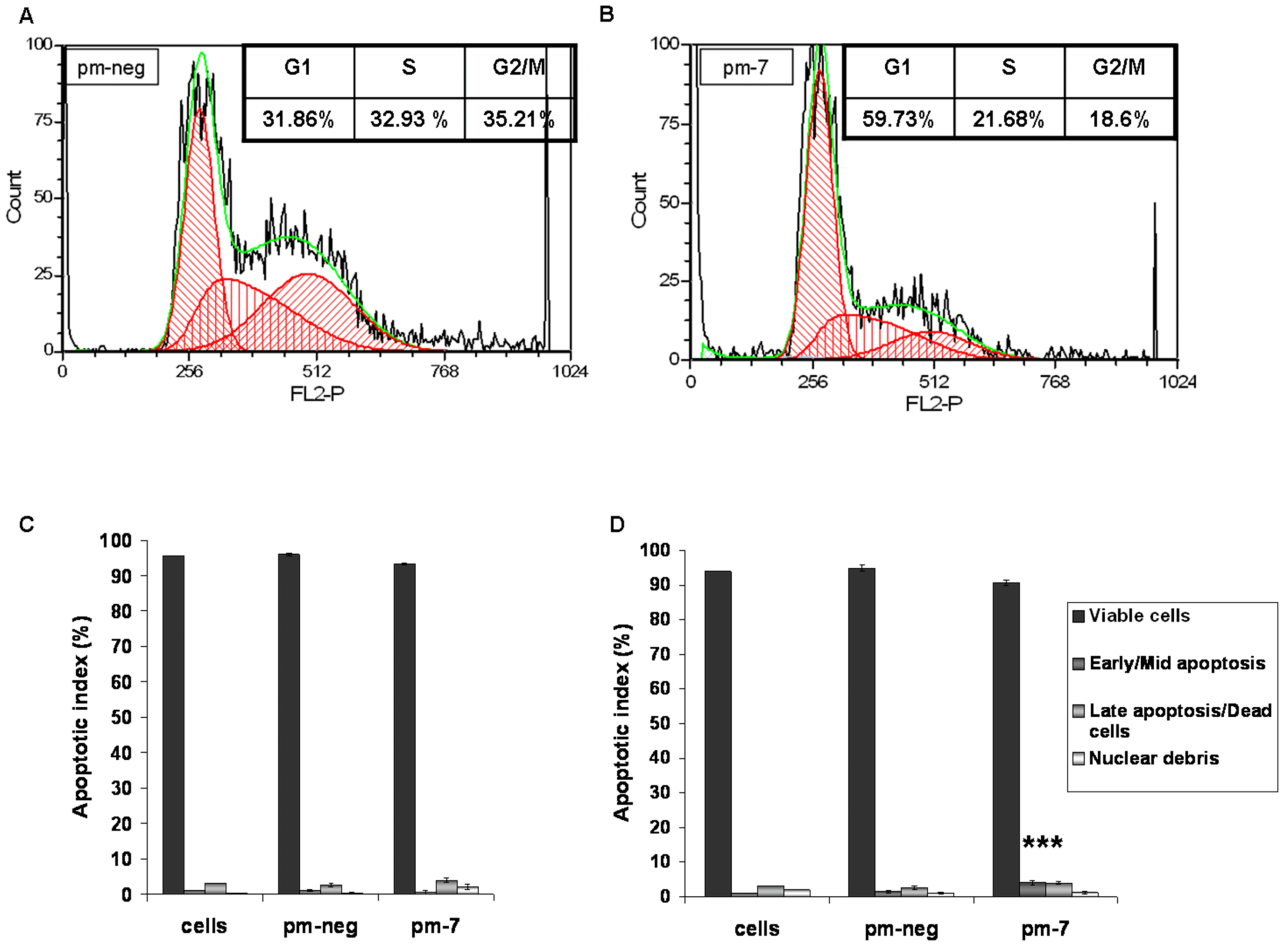
Impact of miR-7 on cell cycle and apoptosis. For cell cycle analysis, cells were stained with Guava Cell Cycle reagent at 3 days after treatment with pm-neg (A) or pm-7 (B). Apoptosis was evaluated with the Nexin assay reagent at day 3 (C) and day 5 (D) after transfection. The data were captured using a Guava Flow cytometer. FCS files from cell cycle assay were extracted and analysed using FCS Express Plus. Standard deviations represent four biological replicates. Significance was evaluated with a Student's t-test. ***: p-value<0.001.

**Figure 3:**
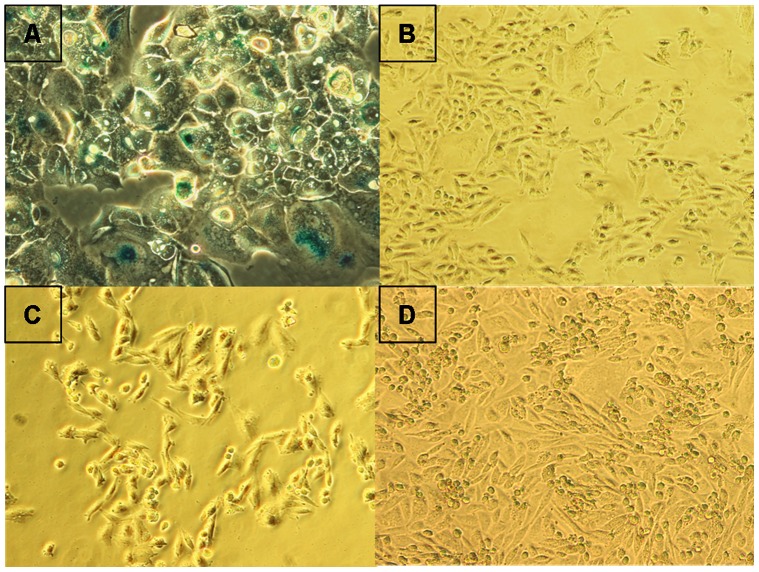
MiR-7 does not induce senescence. β-galactosidase activity was assayed after 96 hrs in cells treated with BrdU or transfected with pm-neg or pm-7. A: HCC1419 cells treated with 50 µM BrdU as positive control for senescence; B: pm-neg-treated CHO cells; C: pm-7-treated cells; D: CHO cells treated with 500 µM BrdU.

Previously, we demonstrated that up-regulation of miR-7 levels induced transient cell growth arrest in CHO cells while maintaining high cell viability [19]. Subsequent to transfection with a miR-7 mimic, cells displayed impaired growth over the following 4 days. The cells subsequently re-entered the cell cycle and proliferated normally (**Fig. 1**). To verify the role of miR-7 in the regulation of cell cycle, we analysed cells 72 hrs after transfection. High levels of miR-7 triggered cell accumulation in the G1 phase thus reducing the proportion of cells in S and G2 (**Fig. 2A&B**). There was no detectable sub-G1 population suggesting that the cells did not undergo apoptosis either in the control or in miR-7 transfected cells (**Fig. 2A&B**). To confirm this we measured apoptosis levels specifically and found that there were no significant changes 72 hrs after transfection (**Fig. 2C**). 120 hrs after transfection there was a small but significant increase in apoptosis in the pm-7 treated cells representing less than 5% of the population (**Fig. 2D**). It is worth noting that at this time point the cells have started to proliferate again as the effects of the transient transfection abate (**Fig. 1**). By way of comparison we investigated the cell cycle distribution of CHO clones over-expressing a miR-7 decoy transcript, effectively depleting endogenous levels of mature miR-7, and found an increase in the percentage of cells in S and G2/M compared to PM-7-treated cells (**Fig. S1**). We also measured the expression of endogenous pre-mir-7 in cells transfected with a miR-7 decoy sponge to check for any feedback loops in response to artificially deregulating the levels of mature miR-7. No change in endogenous expression was observed (data not shown). Thus the high cell viability and the lack of sub-G1 population observed in pm-7 treated cells suggest that high levels of miR-7 do not initiate apoptosis. We investigated the possibility of cellular senescence occurring subsequent to G1 arrest by analysis of SA-β-galactosidase activity. Neither treatment with the DNA intercalating agent, BrDU which causes senescence in replicating cells [20] (and did so in our positive control using HCC1419 breast cancer cells), or transfection with exogenous miR-7 induced SA-β–Gal expression in CHO cells (**Fig. 3**).

### Identification of miR-7 targets

**Figure 4:**
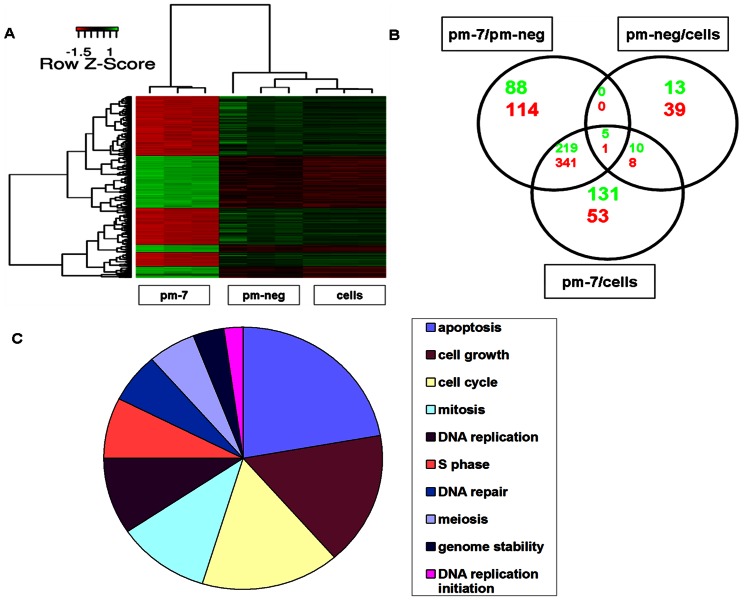
Transcriptomic analysis of miR-7 transfected cells. Following pm-7 or pm-neg transfection, gene expression profiling was performed on biological triplicates using oligonucleotide arrays. Genes were considered to be differentially expressed and statistically significant if a 1.2 fold change in either direction was observed along with a Bonferroni adjusted p-value<0.05. Using the LIMMA method and Bonferroni algorithm, gene expression between the three groups was evaluated and compared (A). Unique and commonly differentially expressed probesets across the three comparisons were identified (B). *Red*: Down-regulated; *Blue*: Up-regulated. The biological processes most significantly represented by these DE genes were identified *in silico* using PANTHER and Pathway Studio (C).

**Figure 5:**
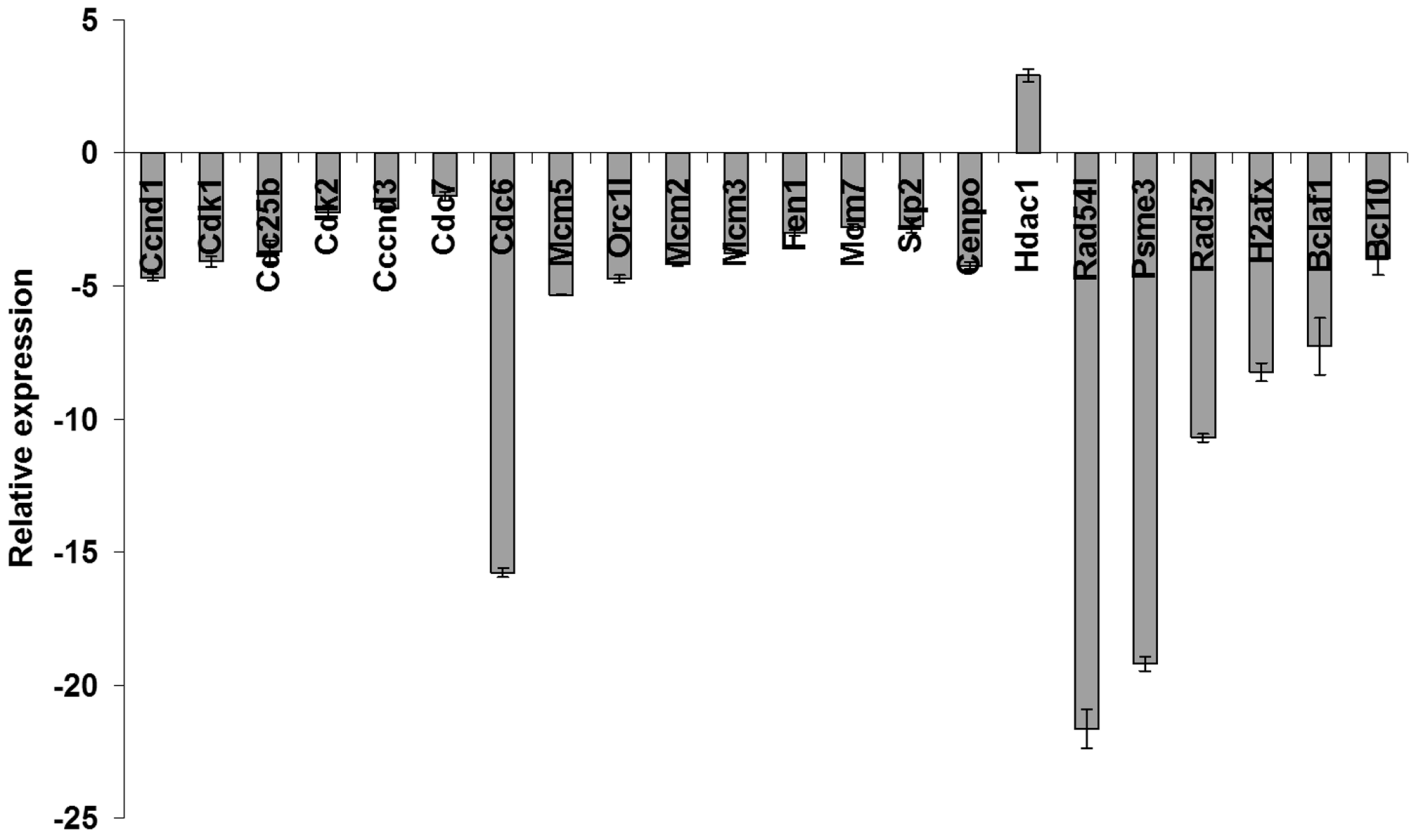
Validation of miR-7 targets. Genes involved in cell cycle regulation and DNA repair were validated by qRT-PCR. Relative expression was calculated using the 2^−ΔΔCt^ method with β-actin used as an endogenous control.

To further investigate the molecular mechanisms underlying this effect and the role of miR-7 in controlling cell growth, we performed gene expression profiling using microarrays. Cells were treated with either a non-specific control (pm-neg) or with mimics of endogenous miR-7 (pm-7). Only a small number of genes were differentially regulated after transfection with pm-neg compared to untreated cells indicating that there was very little non-specific impact of the treatment itself (**Fig. 4A**). In contrast, pm-7 transfection resulted in 341 significantly down-regulated and 219 significantly up-regulated probe sets compared to the controls (**Fig. 4B**). From this group a final list of 355 annotated, non-redundant, differentially expressed gene transcripts was generated. Gene ontology analysis revealed that apoptosis, cell cycle, cell proliferation and DNA repair were the most overrepresented biological processes in this list of genes (**Fig. 4C** **& Table S2**). As we were most interested in genes that were likely direct targets of miR-7 and in order to validate the array data, qRT-PCR was performed on 39 targets from the list of down-regulated genes (**Table S3**). All of these were confirmed to be reduced in pm-7-treated cells, with the exception of HDAC1 which was found to be up-regulated. Among these genes, 22 are involved in the G1/S phase of the cell cycle including Cdk1/2 (down by 4.07-fold and 2.24-fold respectively) and Cyclin D1/3 (down by 4.67-fold and 2.11-fold respectively) (**Fig. 5**). Others are associated with replication, like the MCM family (MCM2, 3, 5 and 7 down by between 2.78-fold and 5.33-fold), or with the DNA damage/repair pathway such as Rad52/Rad54L (down by 10.71-fold and 21.64-fold respectively), Psme3 (down by 19.20-fold) or Bcl-2 associated factor (down by 7.27-fold) (**Fig. 5**, **Table S3**). To further investigate whether the list of differentially expressed genes was enriched for miR-7 targets we performed a one-sided Fishers exact test. The mRNAs found to be down-regulated (n = 199) following miR-7 transfection were, as expected, significantly enriched for TargetScan™ predicted targets (p<0.00002).

### Psme3, Rad54L and Skp2 are direct targets of miR-7

**Figure 6:**
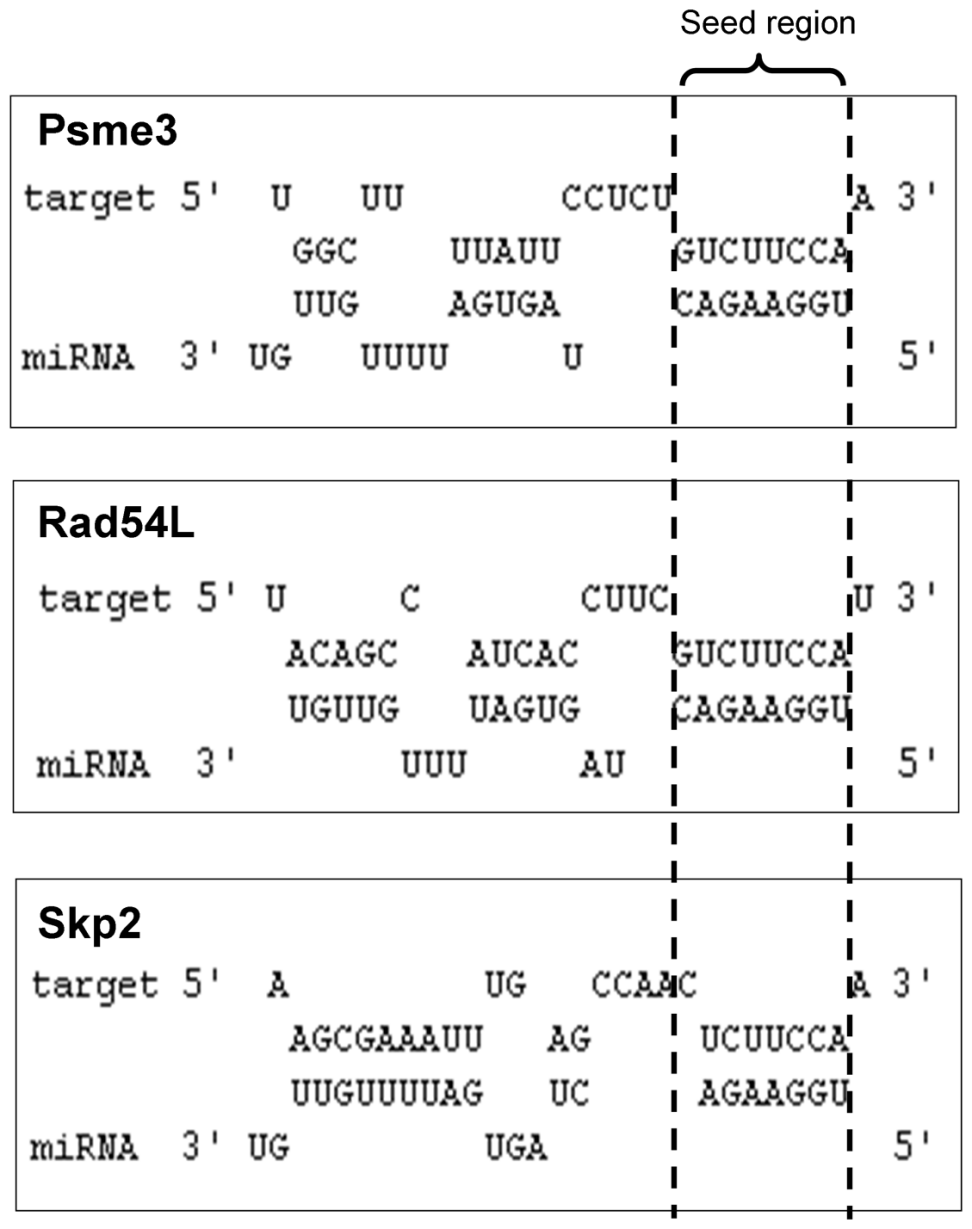
Binding sites of miR-7 predicted in Psme3, Rad54L and Skp2. The mature sequence of cgr-miR-7 was aligned with the sequences of its CHO mRNA targets, Psme3 (A), Skp2 (B) and Rad54L (C) using RNAhybrid [23].

**Figure 7:**
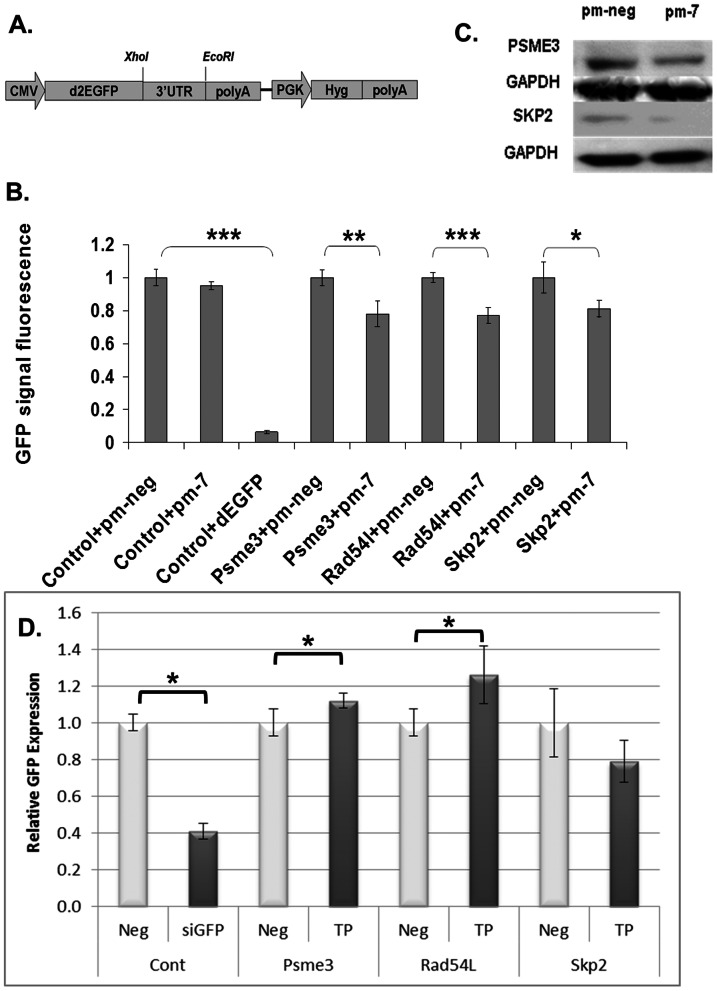
Psme3, Rad54L and Skp2 are direct targets of miR-7. The 3′UTR sequences of Psme3, Skp2 and part of the coding sequence plus the 3′UTR of Rad54L were inserted between *Xho*I and *EcoR*I restriction sites of the CMV-deGFP reporter vector (A). Following co-transfection of 1 µg of reporter with 50 nM of miR-7, GFP fluorescence was analysed using a Guava Easycyte96 system (B). The control was the CMV-deGFP vector. An siRNA against deGFP (dEGFP) was included as a positive control. Levels of endogenous PSME3 and SKP2 proteins were also investigated by western blotting following transfection with PM-Neg or PM-7 (C). Standard deviations represent biological triplicates. Cells were also co-transfected with reporter plasmids (0.5 ug/well in 24w-plate) and 500 nM of site-specific (TP) or control (Neg) target protector oligonucleotides to block miR-7 access to the predicted binding sites. A CMV-GFP vector was co-transfected with non-specific (Neg) or GFP-specific (siGFP) siRNA to ensure transfection efficiency (n = 6) (D). Significance was evaluated with a Student's t-test.*: p-value<0.05; **: p-value<0.01; ***: p-value<0.001.

Having established that several growth and DNA replication-related genes were dysregulated upon miR-7 treatment, we wished to verify whether they were primary or secondary targets of miR-7. Human Psme3 was predicted to have two miR-7 binding sites using Targetscan (Release 6.2), both of which were conserved in the CHO-K1 sequence. Skp2 was predicted to contain a miR-7 site using DIANAmT, miRWalk, miRanda and Pictar5 and again this was found to be conserved in CHO. Rad54L was chosen due to the extent of its dysregulation (downregulated by >20-fold) despite the lack of a predicted canonical miR-7 binding site in its 3′UTR. However, an excellent miR-7 seed binding site was detected just upstream of the stop codon of Rad54L and we were interested to see if this could be responsible for the reduced expression of the gene in the presence of pm-7. This non-canonical site was also conserved across species. The predicted binding interactions between the CHO mRNA sequences of these genes and mature miR-7 (cgr-miR-7) are illustrated in (**Fig. 6**). To complement the array data, expression of these genes was also found to be generally increased in CHO clones with artificially depleted endogenous, mature miR-7 levels (**Fig. S1**). Validation of Psme3, Rad54L and Skp2 as direct targets of miR-7 was performed by co-transfecting cells with pm-7 and reporter plasmids containing the UTR, including part of the coding sequence in the case of Rad54L, downstream of GFP (**Fig. 7A**). The fluorescent signal was significantly reduced for all three targets when exogenous miR-7 was added compared to negative control treated cells (**Fig. 7B**). In addition, endogenous Psme3 and Skp2 levels were confirmed to be down-regulated at the protein level upon pm-7 treatment (**Fig. 7C**) (There was no suitable antibody available to detect Chinese hamster Rad54L). As a further validation we also looked at the impact of blocking the predicted binding sites in the reporter constructs using target protector oligonucleotides (**Fig. 7D**). GFP expression was increased significantly in cells transfected with both Psme3 and Rad54L reporters in the presence of the protector molecules, presumable as a result of reduced access of endogenous miR-7 to the UTR sites. On the other hand there was no increase in expression from the Skp2 UTR reporter.

**Figure 8:**
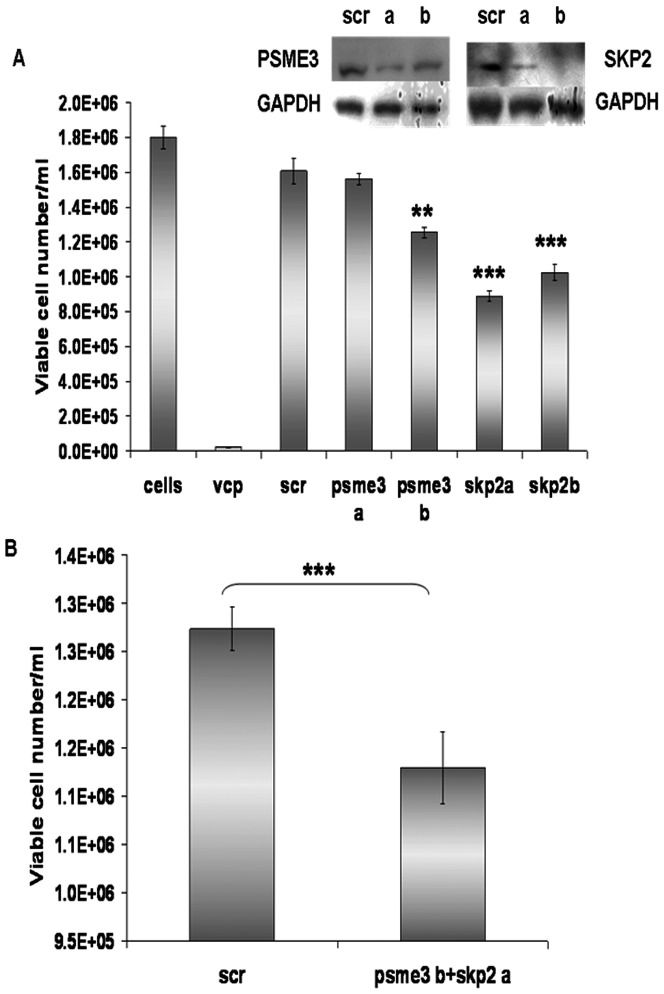
Impact of Psme3 and Skp2 on cell proliferation. Two siRNAs (a&b) for Psme3 and Skp2 were transfected separately (A) or simultaneously (B) at a final concentration of 50 nM. Cell growth was assessed at day 3 after transfection. Knockdown of PSME3 and SKP2 was confirmed by western blotting (A). GAPDH was used as a loading control. Error bars represent standard deviations across biological triplicates. Significance was evaluated with a Sztudent's t-test. **: p-value<0.01; ***: p-value<0.001.

To establish to what extent the phenotypic impact of miR-7 transfection was due to targeting these genes, we depleted Psme3 and Skp2 using siRNAs. Psme3 knockdown led to a 22% reduction in cell density using one of the siRNAs (**Fig. 8A**). Skp2 knockdown reduced cell density by 44% and 36% for the two siRNAs. Neither treatment impacted cell viability. Simultaneous knockdown of Psme3 and Skp2 had only a small but significant impact on cell density (**Fig. 8B**). This observation may be the result of the lower concentration of each co-transfected siRNA used in order to avoid saturation of the cellular RNAi machinery.

### Investigation of other proteins involved in cell growth arrest response and impairment of apoptosis initiation

**Figure 9:**
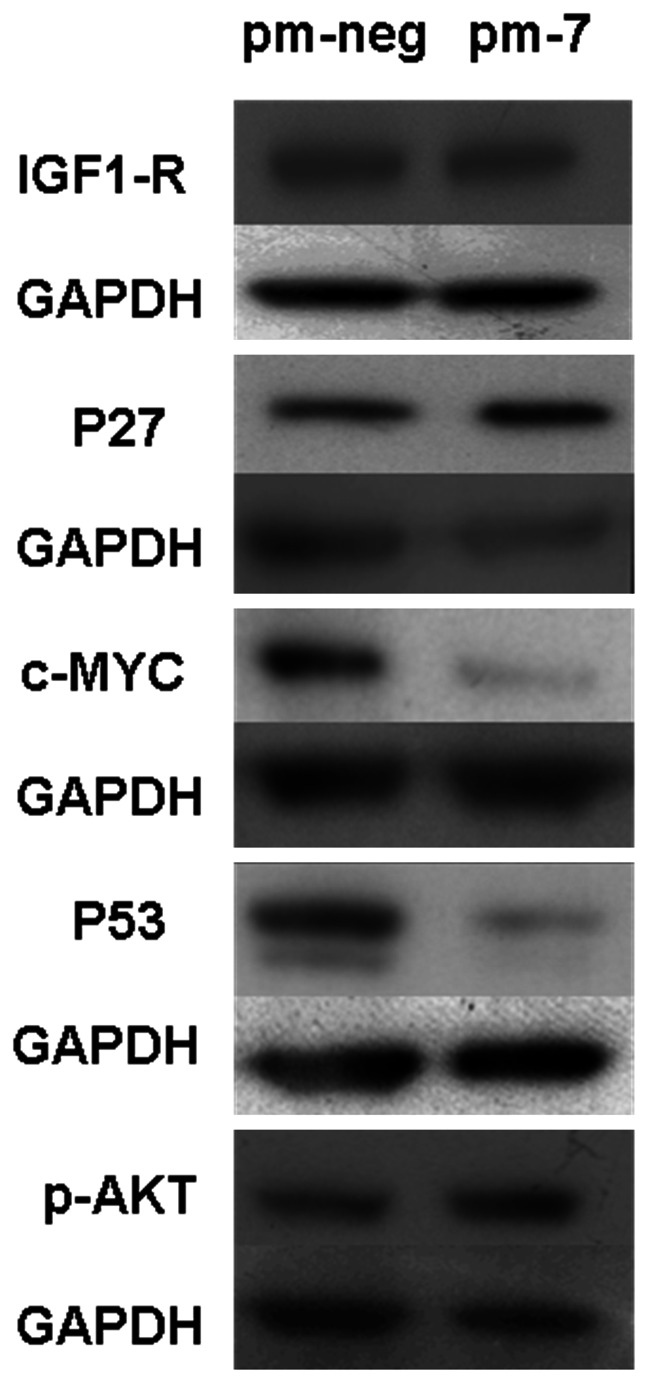
Expression of IGF1-R, p27, c-MYC, p53 and p-AKT in cells transfected with either a non-specific mimic (pm-neg) or miR-7 mimic (pm-7). GAPDH was used as a loading control.

Having identified primary targets of miR-7 binding we were interested to see whether downstream proteins were also affected. Skp2 is known to impact on the abundance of p27^kip1^ and c-Myc, both critical regulators of cell cycle progression [26], [49]. The levels of p27^kip1^ protein were increased after miR-7 transfection (**Fig. 9**). This is consistent with a reduction in Skp2 which would lead to a stabilisation of p27^kip1^ thus impairing G1 to S transition. The proto-oncogene c-Myc was reduced significantly. Skp2 has been reported to interact directly with c-Myc promoting its degradation by ubiquitination thus regulating its turnover at the G1/S transition [21]. Interestingly, p53, a key molecule in cell cycle and apoptosis regulation as well as other processes, was significantly down-regulated. Psme3 is known to promote Mdm2-p53 interaction which triggers ubiquitination and degradation of p53 consequently leading to apoptosis inhibition [22]. One might have expected that the down-regulation of Psme3 would result in an increase in p53 levels, in contrast to what was observed. The levels of p-Akt, which have been shown to rescue cells from apoptosis, were increased after miR-7 transfection suggesting a role of miR-7 in attenuating any apoptotic response. Finally, we found that the levels of IGF1-R protein were not affected despite IGF1-R having previously been shown to be a target of miR-7 in gastric cancer and tongue squamous carcinoma cells [16], [23]. In summary, these results indicate that as well as its direct impact on Psme3, Rad54L and Skp2, miR-7 also influences the cellular levels of critical proteins including p27^kip1^, c-Myc, p53 and p-Akt in order to control cell fate.

## Discussion

Recently there have been several reports on a role for miR-7 in cell proliferation and its dysregulation, particularly down-regulation, leading to various cancers [8]–[12]. We previously showed that following transfection with a miR-7 mimic, CHO cells were growth arrested for 96hrs while maintaining high cell viability and improving production/secretion of a model human glycoprotein [19]. In cancer cells two cell signalling pathways, the epidermal growth factor receptor (EGFR) and the insulin-like growth factor receptor 1 (IGF1-R) signalling, have been reported to be responsible for proliferation inhibition upon miR-7 induction [14], [16], [23]. However, EGFR signalling is not likely to be the pathway by which miR-7 regulates cell proliferation in CHO cells as these cells do not express EGFR [24], although we cannot exclude the possibility that the pathway may still be endogenously active. In addition, we found that there was no difference in IGF1-R expression after miR-7 transfection in CHO cells. To investigate additional mechanisms of action by which miR-7 might control cell growth, we used microarrays to profile miR-7 mimic-treated cells. This identified many genes, including CylinD1/D3, Cdk1/2, Psme3, Rad52/54L and Skp2 that have previously been shown to be dysregulated in cancer. Another interesting observation was that the G1 phase was extended in PM-7-treated cells, a phase that is typically associated with active protein translation. We have previously reported on improved protein production in transgenic CHO cells transfected with a miR-7 mimic [19]. Conversely, we did not observe any enrichment of translation-associated proteins or pathways in the array profiling though proteomic analysis of CHO cells overexpressing miR-7 show depleted ribosomal protein expression [30].

Of the transcripts identified, we focused on three targets, Psme3 (REGγ or PA28γ), Rad54L and Skp2, none of which had previously been shown to be directly regulated by miRNAs but were predicted to bind miR-7 through either their 3′UTR (Psme3, Skp2) or coding sequence (Rad54L) and were representative of the cell proliferation and DNA repair pathways [25], [26], [27].

Although miRNA binding sites in open reading frames are less common than UTR-based sites [28]–[29] the predicted miR-7 target in Rad54L was highly complementary and thus considered worthy of investigation. The reduction in GFP fluorescence observed using the reporter assay indicated a direct interaction between this sequence and miR-7 though the reduction in signal was not as striking as the reduction in endogenous Rad54L mRNA levels observed by qPCR. This may be in part due to the fact that the reporter assay was restricted to only contain this predicted site plus the 3′UTR downstream. There is actually a second predicted miR-7 binding site (in human as well as CHO cells) near the 5′ end of the coding sequence which may also contribute to the regulation of the endogenous transcript. In addition, GFP-based reporter assays tend to be more qualitative than quantitative. Unfortunately, there was no suitable antibody available to establish the levels of Rad54L protein in CHO cells in response to miR-7.

There was strong evidence that both Psme3 and Skp2 were direct targets of miR-7 via their respective 3′UTRs and these results are consistent with proteomic data from our laboratory that showed the down-regulation of Psme3 by 11-fold and 30-fold 48hrs or 96 hours after treatment with pm-7 [30]. However, in the case of Skp2, artificially blocking access to the predicted binding site with morpholinos failed to increase reporter gene expression suggesting that either Skp2 is not a direct target of miR-7 or that the predicted miR-7 binding site in this case is incorrect. Subsequent individual depletion of these genes using siRNA also inhibited cell proliferation, in particular when Skp2 was knocked down. The more modest impact of Psme3 on proliferation might be explained by compensation via redundant proteins in the same family [31], [32] or possibly the less effective depletion of the target by RNAi.

**Figure 10:**
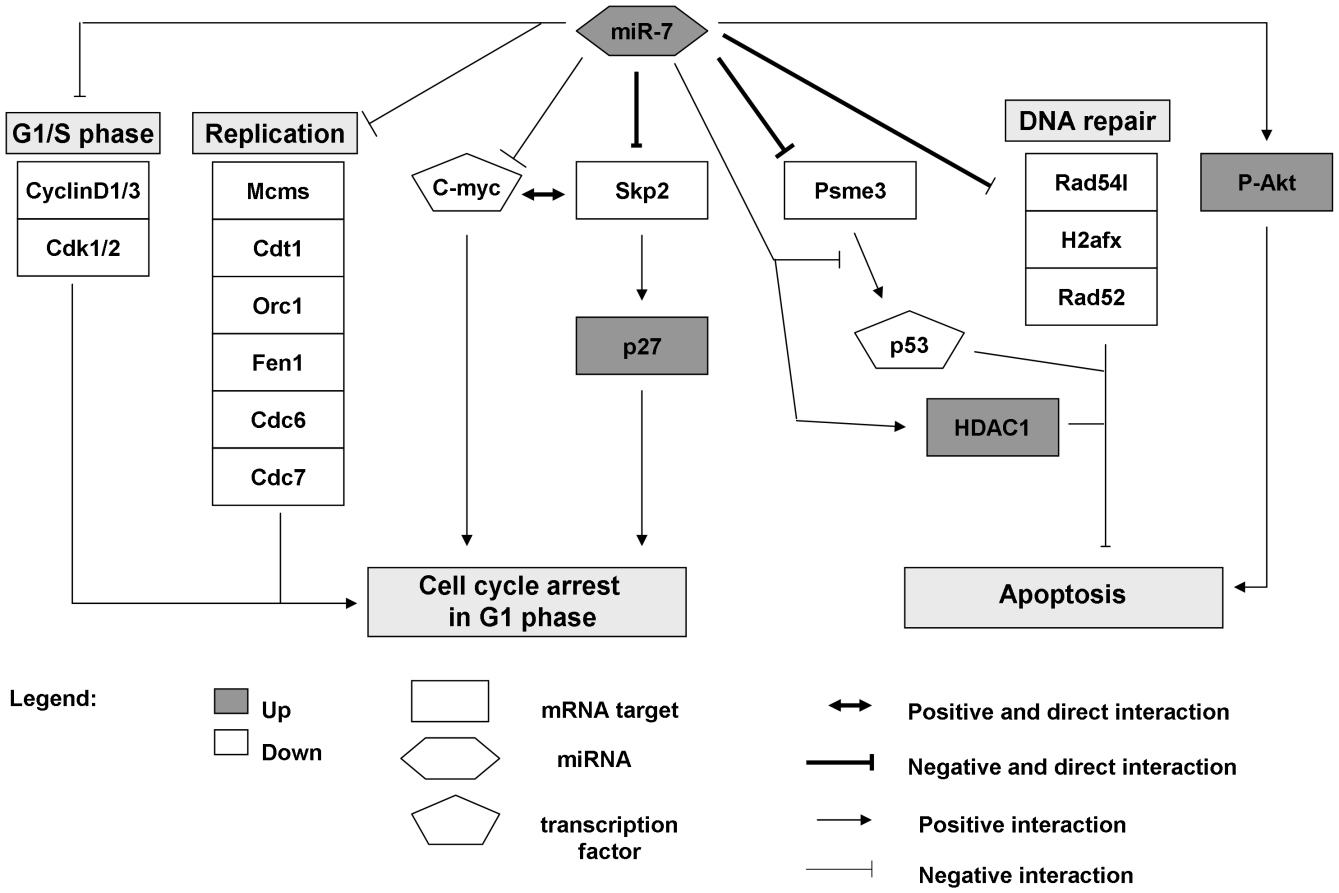
Model network of miR-7 interaction. In purple are the down-regulated mRNA targets and in green the up-regulated mRNA targets. Down-regulated transcription factors are coloured in blue. The bold lines represent direct interactions. The other lines are hypothetical interactions.

The role of Skp2 in miR-7-mediated G1/S block was confirmed by the observation that several of its downstream targets were also dysregulated including an increase in p27^kip1^ and reduced Cks1, Cdk1/2 and CyclinD1/3; all indicative of G1 arrested cell [33], [34], [35]. In addition, the levels of phospho-Akt, another substrate of Skp2, were increased upon miR-7 transfection (**Fig. 10**). Akt is known to play a central role in the regulation of cell proliferation, cell viability and cell cycle arrest in G1 phase by indirect regulation of CyclinD1 inhibition [36], [37]. In contrast to our findings, Skp2 knockdown has been shown to impair activation of Akt in response to ErbB signalling [38], [39]. However, EGFR is not expressed in CHO cells thus the activation of Akt in the presence of reduced Skp2 must be via another pathway. Myc levels have also been reported to be regulated by Skp2 to promote cell cycle progression at the G1/S transition [21], [40] and c-myc is able to activate Skp2 transcription [41]. We showed that the levels of c-myc were dramatically reduced in response to miR-7 upregulation, as would be expected in quiescent cells, which would further re-enforce the down-regulation of Skp2. Interestingly, c-Myc has been reported to be directly regulated by miR-24 through a seedless recognition element [42]. In the same study, high levels of miR-24 induced cell accumulation in the G1 compartment. c-Myc degradation can also be promoted through the recruitment of miR-24 by ribosomal protein L11 [43].

Furthermore the interaction of Skp2 with DNA replication proteins including ORC1, Cdt1 [44], [45] and the observed depletion of other regulators of the S phase including Fen1, cdc6 and the MCM family (MCM2, MCM3, MCM5 and MCM7) would strengthen the block on G1/S transition.

In normal cells, arrest in the G1/S phase and the inhibition of the replication machinery can be initiated by Chk1 or Chk2 in response to DNA damage and frequently results in initiation of apoptosis [46]. However, in our study cells treated with miR-7 remained as viable as the control treated cells. Low Psme3 expression would normally be associated with elevated levels of p53, reduced proliferation and induction of apoptosis [47], [48], [49], [25]. Herein, we found p53 levels to be suppressed despite miR-7-dependent reduction in Psme3, cell growth arrest and no apoptosis. Although miR-7 is acting to arrest cell growth, its role may be to simultaneously ensure that the response is not catastrophic (apoptosis) by directly or indirectly targeting p53 for degradation. Phosphorylation of Akt can also impair apoptosis initiation via subsequent inhibition of pro-apoptotic proteins [50]–[52]. The down-regulation of BCLAF1, a proapoptotic factor that induces p53 and BAX, and triggers down-regulation of MDM2 [53], again points toward a co-ordinating role of miR-7 in preventing apoptosis induction [53], [54]. Likewise, the up-regulation of HDAC, a histone deacetylase involved in p53 degradation [55], [56], [57] is in keeping with the observed response. The low levels of DNA repair proteins including H_2_af, Rad52, Rad54L and Parp1/2 are also likely to moderate any apoptotic response [42].

In conclusion, we suggest that miR-7 has a dual role in CHO cells which counteracts pro-apoptotic signals and activates anti-apoptotic factors to prevent cells undergoing apoptosis-dependent cell death while simultaneously arresting growth in G1 (**Fig. 10**). Taken in this context, miR-7 seems to act as a rheostat to maintain cellular homeostasis. This is supported by previous reports that demonstrated a role of miR-7 in fine-tuning development-specific genes to avoid major cell perturbation and to maintain robustness during environmental perturbations [58], [17].

## Materials and Methods

### Cell culture

CHO-K1 cells expressing human secreted alkaline phosphatase (SEAP) were cultured in 5 ml suspension in spin tubes (CultiFlask, Sartorius) and maintained in serum-free medium, CHO-S-SFMII (#12052-098, Life technologies) at 170 rpm and 37°C with 5% CO2. Cell density and cell viability were measured by flow cytometry using Guava Viacount staining™ (#4000-0040, Guava Technologies Millipore).

### Transient transfection

CHO-K1 SEAP cells seeded at 1×10^5^ cells/ml were transiently transfected with a total concentration of 50 nM mimic molecules (#M-01-D, miRNA mimic, GenePharma), non specific controls (cM-03-D, miRNA mimic negative control, GenePharma) or siRNAs (Custom design, Integrated DNA Technologies) using NeoFx™ transfection reagent (#AM4510, Ambion) in a 2 ml final volume. For the 3′UTR reporter assays, plasmids were complexed with Transit2020 (Mirus) transfection reagent and co-transfected into cells with either 50 nM miRNA mimic or 500 nM Target Protectors (Qiagen). Median GFP expression was measured 24 hrs later on a Guava Easycyte 96 cytometer (Millipore).

### RNA extraction and real time PCR

Total RNA was extracted using Tri-reagent (#T9424, Sigma Aldrich) as recommended by the manufacturer. RNA was reverse transcribed following the High-Capacity cDNA Reverse Transcription protocol (#4368814, Life Technologies) for gene expression assays or using TaqMan® MicroRNA assay protocol (#4324018, Life Technologies) for miR-7 expression analysis (miR-7 # 4427975, snRNU6 endogenous control #4427975, Life Technologies). The cDNA was amplified using SYBR Green mastermix (#4385616, Life technologies) in an AB7500 Real Time PCR instrument and expression analysis was performed using the 2^−ΔΔCt^ method. Primers were designed using DNA oligos design tool (Sigma-Aldrich). The primer sequences are available in Table S1.

### Cell cycle analysis

Cells were washed in 1X PBS, fixed with 200 µl ice-cold 70% ethanol and refrigerated for 12 hours prior to staining. After overnight incubation at 4°C, the cell pellet was washed in 1X PBS, resuspended in 200 µl Guava Cell Cycle reagent containing propidium iodide (#4500-0220, Millipore) and incubated in the dark at 37°C for 15 min before analysis with a Guava Easycyte96 (Millipore). For each step, centrifugation was performed at 1000 rpm for 5 min at room temperature. The data from the FCS files were analysed using MultiCycle™ DNA analysis in the FCS Express 4 flow cytometry software (De Novo software).

### Apoptosis analysis

Cells were harvested and resuspended in 200 µl Guava Nexin reagent (#4500-0450, Millipore) at 72 hrs and 120 hrs after transfection. Cells were incubated for 20 min at room temperature before analysis with a Guava Easycyte96 (Millipore).

### Senescence assay

Cells were fixed 72 hrs after miR-7 transfection or 96 hrs after BrdU addition (#B5002-100MG, Sigma-Aldrich). β -galactosidase activity was assayed as recommended in the senescence β-galactosidase staining kit (#9860S, Cell Signalling).

### Microarray profiling

Cells were seeded at 1×10^5^ cells/ml and transfected with either 50 nM of non-specific control (PM-Neg, Ambion) or miR-7 mimic (PM-7) in a 2 ml serum-free suspension culture. Total RNA extraction was performed 24 hours after transfection using the mirVana miRNA Isolation Kit (#AM1560; Life technologies). RNA quality from biological triplicates was checked using an Agilent 2100 Bioanalyzer (Agilent Technologies). 100 ng of total RNA from each sample underwent cDNA synthesis, followed by cleanup, overnight IVT amplification and labeling using the GeneChip 3′ IVT Express Kit (#901229, Affymetrix) according to manufacturer's instructions. cRNA cleanup was carried out using the GeneChip Sample Cleanup Module (#900371, Affymetrix) according to manufacturer's instructions. The custom CHO oligonucleotide WyeHamster3a microarray (Affymetrix) used in these analyses contains a total of 19,809 CHO-specific transcripts, combining library-derived CHO and publicly-available hamster sequences.

### Data processing and analysis

Microarray data were pre-processed as described [59]. Differential expression analysis was carried out using the R package, LIMMA. [60]. Genes were considered to be differentially expressed upon observation of a 1.2-fold change in either direction along with a Bonferroni adjusted p-value<0.05. The functional relevance of those differentially expressed genes was determined using DAVID bioinformatics suite (http://david.abcc.ncifcrf.gov/).

### 3′UTR cloning

RNA was extracted using TRI reagent (#T9424Sigma Aldrich) and reverse transcribed using the High-Capacity cDNA Reverse Transcription kit (#4368814, Life technologies), with oligo-dT or gene-specific reverse primers. PCR amplicons for Psme3, Rad54L and Skp2 were generated using the Platinium® PCR SuperMix High Fidelity (#12532-016, Life technologies). The PCR program consisted of a denaturation step at 95°C for 2 min followed by 5 cycles of: 95°C for 1 min, 58°C for 30 sec, 72°C for 2 min, and 25 cycles of: 95°C for 1 min, 55°C for 30 sec, 72°C for 2 min, followed by 72°C for 10 min. Primers used were the following (MWG-Eurofins):

Psme3 F: AAAACTCGAGAATCAGTATGTCACCCTACA

Psme3 R: AAAAGAATTCGCAGCTTTAGAAAGAGGTC

Rad54L F: AAAACTCGAGCTTCACCTACAGCCATC

Rad54L R: AAAAGAATTCTCCTGGGCTTACCAATC

Skp2 F: AAAACTCGAGCCAGCTGTGTATGAAGTG

Skp2 R: AAAAGAATTCTTGTTCTTCAAAATCAAGT

The PCR products were restriction enzyme digested and inserted between *Xho*I and *EcoR*I sites in the CMV-d2GFP-XE vector (modified vector derived from pcDNA5-CMV-d2eGFP vector, a kind gift from the Sharp lab, MIT).

### Western blotting

5–20 µg of protein samples were prepared in SDS-PAGE sample buffer, heated at 95°C for 5 min and loaded onto 4–12% NuPAGE Bis-Tris precast gradient gels (#NP0322BOX, Life technologies). Electrophoretic transfer, blocking and development of western blots was carried out as described previously [30]. Membranes were probed with the appropriate primary antibodies (anti-PSME3 #ab97576 Abcam, anti-SKP2 #ab68455 Abcam, anti-c-MYC #ab31426 Abcam, anti-p53, #M 7001Dako, anti-pAKT #92715 cell signalling, anti-p27 #2552S, anti-IGF1R #3027, Cell signalling) diluted in Tris-buffered saline containing 0.1%-Tween 20 (TBS-T). An anti-mouse GAPDH monoclonal antibody (#ab8245, Abcam) was used as a loading control in all experiments.

## Supporting Information

Figure S1 **Stable CHO clones were generated by transfecting parental cells with a plasmid expressing GFP with an artificial UTR containing 4 x miR-7 binding sites downstream.** Control clones contained the same plasmid with a non-specific UTR. The percentage of cells in each phase of the cell cycle was measured by flow cytometry (A,B). Expression of the three predicted target genes was measured by qRT-PCR in three clones from each group. The differences in expression between the non-specific UTR (Cont) and miR-7-binding UTR (Sponge) represent the average of the three clones in each group. Large differences between clones within each group meant that the average difference was not found to be significant (C).
(TIF)

Table S1 **List of Primers used for qPCR.**
(DOC)

Table S2 **Gene Ontology analysis of differentially expressed transcripts in cells transfected with miR-7 mimic compared to control.**
(XLS)

Table S3 **List of genes identified as downregulated upon transfection of cells with miR-7 mimic.** The fold change observed when measured by both array and qPCR is listed as well as whether the gene is a predicted or validated target of miR-7.
(DOC)
