# Supplementary figures and images for: MiR-7 Triggers Cell Cycle Arrest at the G1/S Transition by Targeting Multiple Genes Including Skp2 and Psme3
Source: PLoS One. 2013 Jun 6;8(6):e65671. doi: 10.1371/journal.pone.0065671 (PMC3675065; doi:10.1371/journal.pone.0065671)

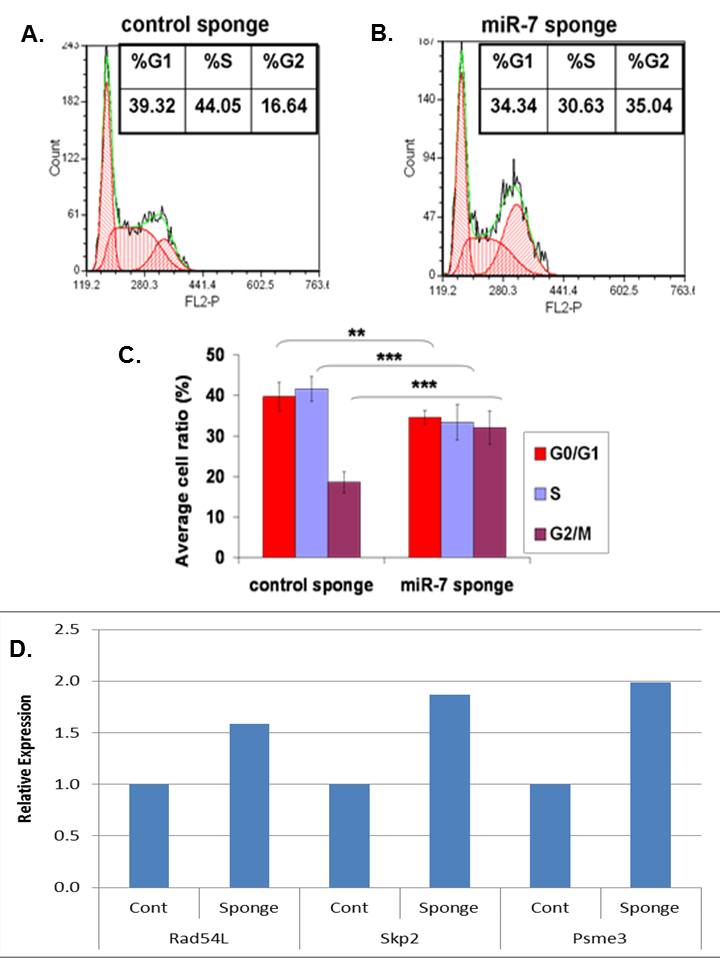

Supplement: Figure S1 — Stable CHO clones were generated by transfecting parental cells with a plasmid expressing GFP with an artificial UTR containing 4 x miR-7 binding sites downstream. Control clones contained the same plasmid with a non-specific UTR. The percentage of cells in each phase of the cell cycle was measured by flow cytometry (A,B). Expression of the three predicted target genes was measured by qRT-PCR in three clones from each group. The differences in expression between the non-specific UTR (Cont) and miR-7-binding UTR (Sponge) represent the average of the three clones in each group. Large differences between clones within each group meant that the average difference was not found to be significant (C). (TIF) [file pone.0065671.s001.tif]
