# Supplementary material for: MiR-7 Triggers Cell Cycle Arrest at the G1/S Transition by Targeting Multiple Genes Including Skp2 and Psme3
Source: PLoS One. 2013 Jun 6;8(6):e65671. doi: 10.1371/journal.pone.0065671 (PMC3675065; doi:10.1371/journal.pone.0065671)
Supplement: Table S1 — List of Primers used for qPCR. (DOC) [file pone.0065671.s002.doc]

Psme3 F: TAT CCC ACC TTG AGC CTG AC

Psme3 R: TGC AGG AAG GAA GGC TAT GT

Apex1 F: ACAGCAAGATCCGCTCCAAA

Apex1 R: GGGCCTAAAGGACACTGGAC

Skp2 F: GGT CCT TTA TGG AGC AAC CA

Skp2 R: CCA CTG CAG ATT CGG AAA AT

Cno F: ACC CAG TTT GGC TGT CTT GA

Cno R: TGC AAT ATG GCA GAG CTG TT

Spata2 F: TCT GGC CCA CAT ACT TCT CC

Spata2 R: GAG TCT GCC CTC TCC AAG TG

Rad54 F: CCA TTA AGA AGC GAG CCA AG

Rad54 R: GGG TTC CAG TCA GGA TCA AA

Ccnd3 F: CCC CAC TAT GGT CAG AGG AA

Ccnd3 R: CCA AGC TCA GTC CCT CAC TC

Peo1 F: ATGTGGAGCAAGCAGCTGGT

Peo1 R: CTCTTCCAGCCGTGTCACAG

Ckap4 F: TGA CTC CAC TTG GCT CAC TG

Ckap4 R: ACA GTG AAG AGC CTG GAG GA

Plp2 F: TCA GGT AGA GGA TGG CTG CT

Plp2 R: TGC TTC AGT GCT TCG ACA TC

Dhfr F: AAT GAC CAC CAC CTC CTC AG

Dhfr R: AGG CAT CGT CCA GAC TTT TG

Setd8 F: GAG GCC ACA TGG TTA ATG CT

Setd8 R: TAA GTG GGG AAT TGG CAG AC

Ccnd1 F: CAC AAC GCA CTT TCT TTC CA

Ccnd1 R: CTC CCT CTG CTT CTC CCT CT

H2afx F: CTG AGG AAA GGC CAC TAT GC

H2afx R: CTT GTT GAG CTC CTC GTC GT

Slc7a5 F: CCA AAG AGC AGG GAC TCT TG

Slc7a5 R: CCA AAA TGC AAA GCA CTG AA

MCM2 F: ACT TCC TAC CTG AGG CAC CA

MCM2 R: ACC TCA GTG AAC GCA ACT CC

Lig1 F: CCC TTT CTC TTT GTC CAC CA

Lig1 R: AGG AGC ATC ACC AGA GCC TA

Orc1l F: TGC CTG ACT CTC TTC CCT GT

Orc1l R: TGC AGA CAG CGT ATG ACC TC

Cdk2 F: AGT GGA GGC ACA ACT TTG CT

Cdk2 R: GTG GAC TGA CGT CAA TGT GG

Aup1 F: TTG TCT GAC GGA GTG AGT CG

Aup1 R: TCC TGG GTC TCA GAA CTG CT

MCM5 F: ACA CTG GCT CCA CTG CTC TT

MCM5 R: TGT GTT TGG ATG CCT GAG AG

MCM3 F: CAG CCT CCA TCA GTG AAA CA

MCM3 R: TGT GTT TGG ATG CCT GAG AG

Aplp2 F: GGCTTATTTGCTGTGCTGGTTT

Aplp2R: TGCTTGGTGACAGCTGGTATAG

Bclaf1 F: CAC CGG AAT ACT GAG GAG GA

Bclaf1 R: TTT TGC AGT GCT AGG CCT TT

Slc39a9 F: GCA GAC AGG TCC CTT GAA AA

Slc39a9 R: GGA CCT GTA CCC AAA CGA GA

Cdc6 F: TCT GTG CCC GAA AAG TAT CC

Cdc6 R: CCG CTG AGA AAC AAG TCC TC

Cnot8 F: ATC AGA GCA AAC CCC CTT CT

Cnot8 R: ATG TGA ATT TTG CCC AGG AG

MCM7 F: AAG TCA AAC CTC GGA TGG TG

MCM7 R: ATT TGG AAC CAC GAG TCT GC

Cenpo F: CTG GTG TCC CTG ATC CAC TT

Cenpo R: CTG ATA TCC AGC GCT TCC TC

Fen1 F: AACCCCGAACCAAGCTTTAG

Fen1 R : GGGCCACATCAGCAATTAGT

Cdc25b F: AGA CGG GTA GCC AAG GTT TT

Cdc25b R: AGC CAC TCT TCA GGT GCA TT

Tfdp1 F: CCA AAC TTC TGG CCA CCT TA

Tfdp1 R: GAC TGC AGC ATC TCC AAT GA

PabpnI F: gtggccatcctaaagggttt

PabpnI R: cgggagctgttgtaattggt

Cdc7 F: CAT CTG ACC TTC CCT CCA AA

Cdc7 R: GAT ACG GCA TGG CAA TAA CC

Rad52 F: GGA GGC CAG AAG GTG TGT TA

Rad52 R: CCC ACG TAG AAT TTG CCA TT

Cdk1 F: CTC CAC CCC TGT TGA CAT CT

Cdk1 R: CGT TGT TAG GAG TCC CCA GA

Bcl10 F: TCT CCT CAG GCA CAC TTC CT

Bcl10 R: CGT GAT CGT AAG GGG AGA AA

Hdac1 F: GGA TGG CCA GAG ACA CTC AT

Hdac1 R: AGC ATC CGG TTT CTG TTA CG
