# Supplementary material for: MiR-7 Triggers Cell Cycle Arrest at the G1/S Transition by Targeting Multiple Genes Including Skp2 and Psme3
Source: PLoS One. 2013 Jun 6;8(6):e65671. doi: 10.1371/journal.pone.0065671 (PMC3675065; doi:10.1371/journal.pone.0065671)
Supplement: Table S3 — List of genes identified as downregulated upon transfection of cells with miR-7 mimic. The fold change observed when measured by both array and qPCR is listed as well as whether the gene is a predicted or validated target of miR-7. (DOC) [file pone.0065671.s004.doc]

| **Gene ID** | **Fold change** | | **qPCR ttest (p<0.05)** | **Prediction algorithm** | **MiRWalk validation** |
| --- | --- | --- | --- | --- | --- |
|  | **Microarray** | **qPCR** |  |  |  |
| **Psme3** | -3.924 | -19.19 | 0.0108 | Diana, miranda, mirwalk, targetscan | Microarray |
| **Apex1** | -3.493 | -2.510 | 0.0430 | No |  |
| **Skp2** | -3.267 | -2.739 | 0.0138 | Diana |  |
| **Cno** | -3.236 | -3.943 | 0.0017 | Diana, miranda, mirwalk,targetscan |  |
| **Spata2** | -3.167 | -4.747 | 0.0097 | Diana, miranda, miRwalk, pictar, targetscan | Sequencing |
| **Rad54L** | -3.148 | -21.642 | 0.0478 | Mirwalk, pictar |  |
| **Ccnd3** | -3.137 | -2.109 | 0.0034 | No |  |
| **Peo1** | -2.874 | -8.863 | 0.0019 | Diana |  |
| **Ckap4** | -2.871 | -2.828 | 0.0374 | Diana,miranda, mirwalk, pictar, targetscan | Microarray, sequencing |
| **Plp2** | -2.815 | -4.569 | 0.0027 | Diana, miranda, mirwalk, pictar, targetscan |  |
| **Dhfr** | -2.764 | -5.126 | 0.0266 | No |  |
| **Setd8** | -2.667 | -2.648 | 0.0038 | Diana, miranda, pictar, targetscan |  |
| **Ccnd1** | -2.555 | -4.669 | 0.0003 | No | Microarray, qPCR |
| **H2afx** | -2.552 | -8.238 | 0.0092 | No |  |
| **Tmem55b** | -2.510 | -3.493 | 0.0005 | No |  |
| **Slc7a5** | -2.506 | -4.710 | 0.0140 | No | Reporter |
| **MCM2** | -2.493 | -4.190 | 0.0132 | No |  |
| **Lig1** | -2.485 | -5.998 | 0.0074 | No |  |
| **Orc1l** | -2.424 | -4.730 | 0.0161 | No |  |
| **Cdk2** | -2.413 | -2.245 | 0.0252 | MiRwalk |  |
| **Aup1** | -2.399 | -3.118 | 0.0002 | No |  |
| **MCM5** | -2.391 | -5.332 | 0.0310 | No |  |
| **MCM3** | -2.251 | -3.765 | 0.0001 | No |  |
| **Aplp2** | -2.166 | -5.207 | 0.0043 | No | Microarray |
| **Bclaf1** | -2.005 | -7.266 | 0.0001 | No |  |
| **Slc39a9** | -1.963 | -3.434 | 0.0286 | No |  |
| **Cdc6** | -1.861 | -15.770 | 0.0006 | No |  |
| **Cnot8** | -1.851 | -2.622 | 0.0160 | Diana, miranda, pictar, targetscan | Microarray, sequencing |
| **MCM7** | -1.847 | -2.779 | 0.0317 | No |  |
| **Cenpo** | -1.783 | -4.238 | 0.0002 | Diana, miranda |  |
| **Fen1** | -1.767 | -2.997 | 0.0012 | No |  |
| **Cdc25b** | -1.752 | -3.706 | 0.0481 | No | Microarray |
| **Tfdp1** | -1.544 | -2.624 | 0.0079 | Miranda |  |
| **Pabpn1** | -1.311 | -2.005 | 0.0002 | Diana |  |
| **Cdc7** | -1.244 | -1.625 | 0.0305 | No |  |
| **Rad52** | -0.851 | -10.708 | 0.0020 | No |  |
| **Cdk1** | -0.754 | -4.071 | 0.0084 | Diana, targetscan |  |
| **BCL10** | -0.737 | -3.968 | 0.0069 | No |  |
| **Hdac1** | -2.529 | 2.920 | 0.0108 | Diana, targetscan |  |
